# Environmental exposures and intergenerational cardiovascular risk: epigenetic reprogramming via parental exercise

**DOI:** 10.3389/fphys.2026.1932608

**Published:** 2026-10-01

**Authors:** Yuhang Zheng, Guanfeng Qin, Zijian Li, Xiaoying Zhu, Haiwang Shi, Fulong Zhang

**Affiliations:** 1 Laboratory of Regenerative Medicine in Sports Science, School of Physical Education and Sports Science, South China Normal University, Guangzhou, China; 2 Innovation Center for Integration of Sports and Medicine, Guangdong Medical University, Dongguan, China

**Keywords:** cardiovascular disease, cardiovascular exposome, epigenetic regulation, intergenerational effects, parental exercise

## Abstract

Cardiovascular disease (CVD) remains a predominant cause of global mortality. The spectrum of traditional risk factors is rapidly expanding, largely driven by the modern cardiovascular exposome. This comprehensive exposome encompasses environmental hazards, sedentary lifestyles, and severe psychosocial stress. Clinical management has evolved toward an integrated system to counteract these threats, yet traditional strategies primarily target the middle and late stages of pathogenesis. Addressing this limitation, modern research has shifted its focus to intergenerational effects. At the molecular level, epigenetic regulation may constitute a key mechanistic link between parental environmental exposures and cardiovascular susceptibility in offspring. Harmful parental exposures can reshape epigenetic regulation within germ cells placental tissues and the developing fetus. By contrast regular physical activity prior to or during pregnancy may exert favorable effects on these biological processes as well as the intrauterine environment conferring potential protective benefits to offspring cardiovascular health. Consequently, future strategies for CVD prevention and control must adopt a life-course perspective. The proactive optimization of the epigenetic environment during early life holds the potential to reduce the burden of CVD at its source.

## 1 Introduction

Cardiovascular disease (CVD) remains a major global public health challenge. According to the World Health Organization, CVD consistently ranks as the leading cause of death worldwide, responsible for approximately 17.9 million annual deaths and accounting for 32% of all global mortality (World Health Organization, 2025). Despite substantial advances in CVD prevention and therapeutic strategies in recent decades, this condition continues to impose severe health threats and exert substantial socioeconomic burdens across the globe. Furthermore, evidence from the Global Burden of Disease studies highlights CVD as a predominant driver of global disability-adjusted life year loss.

Cardiovascular disease not only reduces healthy life expectancy but also induces long-term disability, including persistent functional limitations stemming from heart failure and post-stroke sequelae. These chronic complications substantially compromise individual well-being and overall quality of life (Vos et al., 2020). Although existing clinical risk factors remain of significant value, their individual effects fail to fully account for the total cardiovascular risk. Researchers define this hidden danger as residual cardiovascular risk (Al-Kindi et al., 2023). Environmental exposures and modern lifestyle changes heavily drive this residual risk. To understand this burden, modern researchers use the concept of the cardiovascular exposome (Münzel et al., 2023). The exposome represents the sum of all environmental exposures an individual faces throughout their lifetime. These external factors include environmental hazards, lifestyle factors, and psychosocial stress. The exposome framework moves beyond analyzing single risk factors. It explores how multiple environmental exposures interact to shape cardiovascular health and disease across the life course. Importantly, the impacts of such exposures are not confined to the directly exposed individual, given that parental environmental and metabolic states correlate with cardiovascular and metabolic susceptibility in offspring. Epigenetic regulation may constitute a key biological link within this process by bridging parental exposures to molecular alterations in germ cells, placental tissues, and the developing fetus. These findings further raise the possibility that the preventive window for CVD extends to the pre-conception and gestational periods. Parental exercise constitutes a potentially modifiable factor that can modulate intrauterine and placental environments alongside sperm-associated molecular profiles, with downstream implications for offspring cardiovascular development. Accordingly, this review addresses the cardiovascular exposome from a life-course and intergenerational perspective, placing particular emphasis on epigenetic programming and the putative protective role of parental exercise for offspring cardiovascular health.

## 2 The cardiovascular exposome and modern risk factors

Traditional cardiovascular risk factors include hypertension, dyslipidemia, diabetes mellitus, tobacco smoking, and obesity. However, rapid urbanization and environmental transitions have reshaped this conventional risk paradigm. The coronavirus disease 2019 (COVID-19) pandemic further underscored this shift. This global health crisis has offered novel insights into the cardiovascular exposome, which demonstrated how different environmental exposures interact to trigger cardiovascular injury.

COVID-19-associated cardiovascular risks extend far beyond the acute phase and persist long after patient recovery (Xie et al., 2022). The elevated risks arise from both direct viral toxicity and pandemic-related lifestyle and mental health disturbances. As a direct external pathogenic hazard, severe acute respiratory syndrome coronavirus 2 (SARS-CoV-2) invades cardiomyocytes and endothelial cells via binding to the angiotensin-converting enzyme 2 (ACE2) receptor. This process triggers microcirculatory dysfunction and systemic inflammation, thereby elevating the risks of viral myocarditis and thrombosis (Šikić et al., 2021). Quarantine and movement restrictions have reduced physical activity and disrupted dietary habits, disturbing metabolic homeostasis and exacerbating CVD risk (Martinez-Ferran et al., 2020). Meanwhile, social isolation and anxiety have led to sleep disorders and depression, which drive neuroendocrine and immune disorders, exerting sustained harm to the cardiovascular system (Seighali et al., 2024).

In summary, the pandemic clearly highlighted three pivotal components of the cardiovascular exposome: environmental hazards, lifestyle factors, and psychosocial stress. Building on this observation, the following section broadens the discussion beyond the pandemic context. Through analyzing epidemiological evidence and biological mechanisms, this review seeks to construct a systematic, multidimensional framework for understanding modern CVD risks (**Figure 1**).

**Figure 1:**
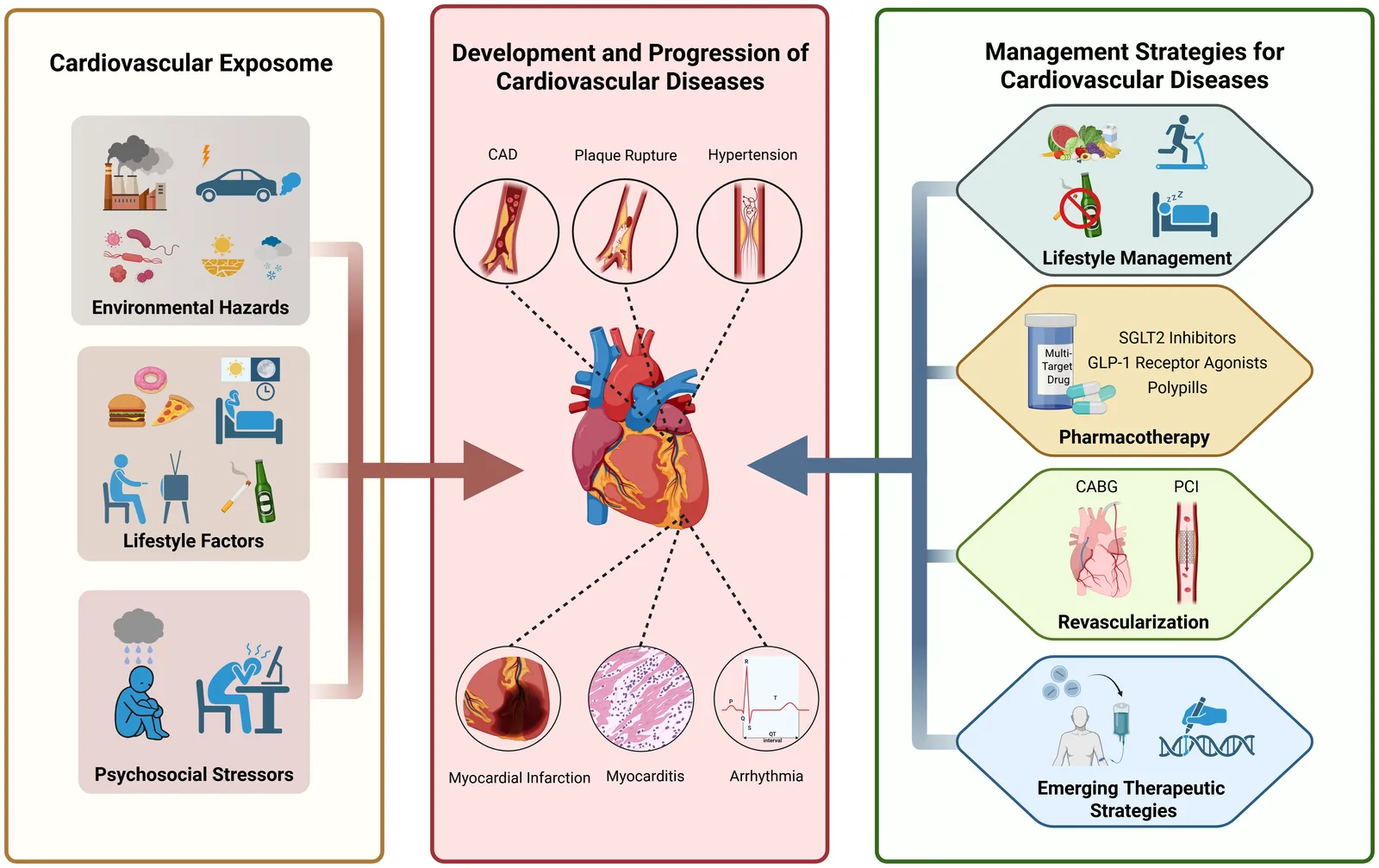
The cardiovascular exposome and CVD management strategies. The modern cardiovascular exposome includes environmental hazards, lifestyle factors, and psychosocial stress. These adverse factors synergistically disrupt physiological homeostasis, driving the pathogenesis and progression of CVD. To counteract this progression, CVD management strategies currently integrate lifestyle management, pharmacotherapy, revascularization, and emerging therapeutic strategies. Created using biorender.com.

### 2.1 Environmental hazards

Physical and chemical environments profoundly modulate cardiovascular health. Rapid industrialization and urbanization expose human populations to a complex spectrum of environmental hazards. These persistent threats comprise pathogenic microbes, heavy metals, traffic pollutants, noise, and extreme temperature fluctuations. Instead of acting in isolation, these diverse environmental stressors promote CVD pathogenesis through two synergistic biological mechanisms the direct toxic-injury pathway and the neuroendocrine stress pathway (Al-Kindi et al., 2020).

The direct biological toxicity pathway occurs when environmental hazards enter the systemic circulation through inhalation, ingestion, or skin contact. These environmental hazards, including pathogens, heavy metals, and traffic exhaust, directly attack cardiovascular tissues and trigger severe oxidative stress alongside widespread inflammatory cascades. Prolonged exposure to multiple chronic pathogens triggers immune cross-reactivity via molecular mimicry. This reaction causes the body to mistakenly attack its own cardiovascular tissues (Tschöpe et al., 2020). Meanwhile, chemical pollutants like heavy metals and traffic exhaust disrupt essential cellular functions. They rapidly deplete antioxidant reserves, disrupt calcium signaling through an “ionic mimicry” mechanism, and block mitochondrial energy production (Pan et al., 2024; Wāng et al., 2025). Certain gaseous pollutants, including carbon monoxide (CO), further exacerbate cardiovascular damage by impairing systemic oxygen transport and, at high exposure concentrations, accelerating thrombotic complications (Chu et al., 2021; Cho et al., 2024). This direct cellular toxicity compromises the structural integrity of the heart and blood vessels.

Beyond inflicting direct physical damage on the cardiovascular system, environmental hazards perturb neuroendocrine function. Physical stressors such as traffic noise and extreme temperature fluctuations, together with specific chemical pollutants, serve as persistent triggers of nervous-system activation (Münzel et al., 2021a; De Vita et al., 2024). Such stimuli drive hyperactivation of the hypothalamic-pituitary-adrenal (HPA) axis and the sympathetic-adrenal-medullary (SAM) axis, facilitating stress-hormone release. This neuroendocrine dysregulation acutely modulates hemodynamics through elevated heart rate, peripheral vasoconstriction and raised blood pressure, which in turn increases myocardial oxygen consumption (Osborne et al., 2020). Sustained neuroendocrine disturbance remodels cardiac energy metabolism and accelerates pathological ventricular remodeling, markedly heightening atherosclerotic plaque vulnerability (Marques et al., 2025; Topriceanu et al., 2025). These findings further support the inclusion of air pollution and related environmental stressors within the cardiovascular exposome, through which environmental exposures may influence cardiovascular health via multiple biological pathways (Juarez et al., 2026).

The direct toxic-injury pathway and the neuroendocrine stress pathway do not function in isolation but interact continuously to amplify pathological outcomes. Extreme weather events frequently synergize with severe air pollution. Joint exposure to ozone and heat waves has been associated with an increased risk of cardiovascular mortality (Xu et al., 2023). Such combined effects establish a solid pathophysiological basis for environmentally mediated CVD.

### 2.2 Lifestyle factors

Individual behavioral choices profoundly modulate cardiovascular health. Contemporary human behavioral patterns are typified by ultra-processed food consumption, circadian rhythm disruption, marked physical inactivity, and misuse of addictive substances. Such behavior-related systemic disturbances compromise vascular endothelial defensive mechanisms by triggering metabolic inflammation, oxidative stress, and autonomic dysfunction. Notably, these endogenous disturbances act synergistically with external environmental hazards such as air pollution, substantially accelerating CVD progression.

Modern dietary habits often displace fiber-rich natural foods with ultra-processed products high in sugar, sodium, trans-fatty acids, and industrial additives. High intake of ultra-processed foods shows a significant positive linear association with CVD mortality (Gauci et al., 2025). This dietary pattern severely compromises intestinal barrier integrity through the induction of gut microbiota dysbiosis. Increased gut permeability enables endotoxins such as lipopolysaccharides and metabolites including trimethylamine N-oxide to enter the systemic circulation. This event drives low-grade systemic inflammation promotes foam-cell formation and accelerates thrombogenesis (Kondapalli et al., 2025).

Favorable sleep characteristics are critical for sustaining cardiovascular homeostasis, whereby sleep duration, quality, and regularity are all associated with cardiovascular outcomes. Insufficient sleep directly blunts nocturnal cardiovascular recovery. It sustains sympathetic nervous system activation, leading to pathological nocturnal elevations in blood pressure and heart rate. Sleep deprivation further activates the nuclear factor-κB (NF-κB) pathway to drive release of pro-inflammatory cytokines such as interleukin-6 (IL-6) and C-reactive protein, thereby exacerbating vascular endothelial dysfunction (Irwin et al., 2015). Poor sleep quality, frequently attributable to obstructive sleep apnea (OSA), produces recurrent apnea-hypopnea cycles. Severe intermittent hypoxia under these conditions generates large quantities of reactive oxygen species (ROS) and precipitates systemic inflammation (Kohler and Stradling, 2010). Irregular sleep-wake cycles trigger physiological desynchronization between the central hypothalamic circadian clock and peripheral clocks within cardiovascular tissues. This desynchronization perturbs core clock genes including circadian locomotor output cycles kaput (CLOCK) and brain and muscle ARNT-like 1 (BMAL1). It disturbs the secretion of metabolic enzymes and raises thrombotic risk by modifying platelet aggregability (Kelters et al., 2025).

Physical inactivity and prolonged sedentary behavior constitute independent, highly prevalent cardiovascular risk factors. Prolonged sitting may attenuate contraction-responsive myokine signaling, potentially reducing some of the anti-inflammatory and cardiometabolic benefits associated with skeletal-muscle activity (Ferreira-Santos et al., 2024). Meanwhile, prolonged sitting markedly reduces leg blood flow, thereby diminishing shear-stress stimulation of the vascular endothelium, which may reduce endothelium-derived nitric oxide production and impair vascular function (Fujie et al., 2026).

Addictive substances chiefly tobacco products, electronic cigarettes and alcohol exert broad detrimental effects on cardiovascular health. Tobacco smoke and e-cigarette aerosols elicit robust oxidative stress via nicotine and aldehyde constituents. These compounds substantially reduce nitric oxide bioavailability, driving vascular endothelial dysfunction and atherosclerotic plaque instability. Sustained sympathetic nervous-system activation by nicotine provokes vasospasm and pronounced hemodynamic swings that predispose to arrhythmias (Zong et al., 2024). Alcohol consumption confers substantial independent cardiotoxicity, and recent epidemiological evidence rejects the existence of any safe intake threshold or putative cardioprotective effects (Piano et al., 2025). Ethanol and its metabolite acetaldehyde directly perturb myocardial calcium homeostasis and the mitochondrial respiratory chain. Such disturbance hinders synthesis of myocardial contractile proteins and promotes interstitial fibrosis, establishing the pathological substrate for alcoholic cardiomyopathy (Piano and Phillips, 2014). Acute heavy alcohol intake shortens the atrial effective refractory period and slows conduction velocity, generating an arrhythmogenic milieu that underlies the clinical entity of “holiday heart syndrome”.

### 2.3 Psychosocial stressors

Psychological stressors including chronic stress, depression, social isolation, and loneliness constitute independent risk factors for CVD. These pathological stimuli converge on the brain-heart axis. Chronic psychological distress drives sustained metabolic hyperactivation within the amygdala. Via the neural-hematopoietic axis, such hyperactivation promotes proliferation of bone-marrow-derived hematopoietic stem cells and fosters enhanced release of pro-inflammatory cytokines into the circulation. This cytokine surge provokes systemic vascular inflammation and atherosclerotic plaque destabilization. Acting in concert with sustained HPA axis and sympathetic nervous-system activation, these processes translate psychological stress into tangible organic cardiovascular injury (Levine et al., 2021).

Such heterogeneous stressors compromise cardiovascular function by disrupting allostatic load across neuroendocrine and immunological pathways. Persistent psychological burden disturbs catecholamine and cortisol homeostasis, precipitating tachycardia, raised blood pressure, visceral adiposity, and marked insulin resistance. Concurrently, these stressors substantially suppress eNOS activity, thereby exacerbating vascular endothelial inflammation and atherosclerotic progression (Vaccarino and Bremner, 2024). This autonomic dysregulation is particularly evident during depressive episodes, where reduced heart-rate variability and blunted baroreflex sensitivity raise susceptibility to life-threatening arrhythmias (Apostolos et al., 2025). At the cellular level, psychological stress remodels immune and metabolic responses. Social isolation triggers the conserved transcriptional response to adversity, upregulating pro-inflammatory genes in leukocytes as well as toll-like receptor 4 (TLR4) expression in monocytes, which in turn disturbs lipid metabolism (Cole, 2019; Haj-Mirzaian et al., 2021).

## 3 CVD management strategies

Given the far-reaching influence of the cardiovascular exposome, contemporary preventive and therapeutic strategies are undergoing a broad shift from single-target interventions toward the development of a multi-tiered, integrated risk-management framework (Visseren et al., 2021). This overarching framework covers diverse therapeutic dimensions, extending from lifestyle modification to molecular-targeted regulation (**Figure 1**).

### 3.1 Lifestyle management

#### 3.1.1 Dietary pattern optimization

Optimising dietary patterns serves as the foundational interventional strategy for CVD prevention. It fosters a favorable metabolic internal environment for the cardiovascular system. Modern clinical nutrition has transitioned from restricting specific nutrients to reshaping holistic dietary patterns that promote cardiac health. Among dietary patterns with established cardiovascular benefits, the Mediterranean and Dietary Approaches to Stop Hypertension (DASH) diets have been extensively studied (Van Horn et al., 2016). The Mediterranean diet, rich in polyphenolic antioxidants and monounsaturated fatty acids, directly targets chronic vascular inflammation and regulates lipid metabolism. This diet exhibits notable advantages in stabilizing atherosclerotic plaques and reducing all-cause mortality (Laffond et al., 2023). By contrast, the DASH diet, characterized by high potassium and low sodium intake, promotes urinary sodium excretion and enhances the bioavailability of nitric oxide. These mechanisms significantly improve blood pressure and reduce ventricular afterload (Chaudhary and Wainford, 2020). In individualized clinical interventions, the DASH diet is preferentially recommended for patients prioritizing blood pressure management. Meanwhile, the Mediterranean diet demonstrates superior interventional potential in regulating lipid metabolism, mitigating vascular inflammation, and preventing long-term cardiovascular events (Karam et al., 2023).

#### 3.1.2 Scientific exercise prescriptions

Regular physical exercise serves as a cornerstone of cardiovascular prevention and rehabilitation, with well-documented benefits for endothelial function. To circumvent the risk of acute cardiovascular events triggered by strenuous physical activity, modern cardiac rehabilitation systems no longer blindly encourage patients to simply increase exercise volume. Instead, they emphasize the necessity of professional medical evaluations to formulate scientific, safe, and personalized exercise prescriptions (Franklin et al., 2020).

In cardiac rehabilitation, high-intensity interval training (HIIT) can improve cardiorespiratory fitness, including peak oxygen uptake (VO_2peak_), in appropriately selected patients (McGregor et al., 2023). For patients at increased risk of arrhythmias, however, HIIT should be prescribed with appropriate exercise testing and clinical monitoring. Additionally, resistance exercise effectively improves the basal metabolic rate, insulin sensitivity, and body composition by increasing skeletal muscle mass and strength, thereby indirectly alleviating the cardiovascular metabolic burden. For healthy adults and many individuals with stable CVD, current guidelines recommend regular aerobic exercise totaling at least 150 minutes of moderate-intensity activity per week, with resistance training incorporated according to individual clinical status and exercise goals (Pelliccia et al., 2021). Exercise prescriptions for individuals with CVD should nevertheless be tailored according to disease characteristics, functional capacity, and the risk associated with exercise.

#### 3.1.3 Smoking cessation and alcohol restriction

Sustained exposure to exogenous toxic substances can readily offset the reparative gains conferred by dietary modification and physical exercise. Smoking cessation and alcohol limitation mitigate ongoing injury to the vascular endothelium, myocardial electrophysiological function, and systemic inflammatory status at its source, yielding considerable cardiovascular health benefits. Smoking cessation markedly lowers circulating carbon monoxide and pro-inflammatory cytokine levels, thereby ameliorating impaired endothelium-dependent vasodilation and slowing atherosclerotic progression. Relative to persistent smokers, individuals who achieve smoking cessation attain substantial reductions in CVD risk within five years. Moreover, smoking cessation initiated at any time point is associated with substantially improved cardiovascular outcomes and enhanced survival (Duncan et al., 2019; Mesnier et al., 2025). Mere reduction in smoking quantity, however, does not produce meaningful cardiovascular benefits for affected patients. Within clinical settings, pharmacologically supported smoking-cessation therapies combined with behavioral counselling help to maintain long-term abstinence and further lower cardiovascular risk (Nazir et al., 2025).

With respect to alcohol limitation, contemporary medical evidence clearly demonstrates that any alcohol intake increases risks of arrhythmia and premature mortality (Ortolá et al., 2024). Accordingly, the dominant public-health consensus advises habitual drinkers to reduce consumption, discourages non-drinkers from initiating alcohol use, and identifies complete abstinence as the safest intake level for patients with CVD. In clinical practice, healthcare practitioners should implement universal alcohol-use screening for patients. For individuals diagnosed with alcohol-use disorder, comprehensive management protocols combining neurotransmitter-modulating agents such as naltrexone with psychosocial interventions are required to attenuate craving and prevent relapse (Kranzler and Soyka, 2018).

#### 3.1.4 Stress management and sleep health

Beyond rectifying the direct damage inflicted by external chemical toxins like nicotine and alcohol, modern cardiovascular management emphasizes integrating stress intervention and sleep management into routine therapeutic frameworks to restore internal neuroendocrine homeostasis (Levine et al., 2021). Psychosocial interventions, including cognitive behavioral therapy, mindfulness meditation, and yoga, may improve maladaptive cognitions and stress-related symptoms. These interventions optimize autonomic nervous system function, significantly improve heart rate variability, stabilize blood pressure fluctuations, and reduce myocardial oxygen consumption, thereby effectively lowering mortality from incident CVD and rates of non-fatal recurrence (El-Malahi et al., 2024; Wu et al., 2026).

High-quality nocturnal sleep is a critical window for the self-repair of the cardiovascular system. The American Heart Association has officially incorporated sleep health into its core cardiovascular health intervention framework (Life’s Essential 8), expanding the focus from mere sleep duration to multi-dimensional sleep health (Lloyd-Jones et al., 2022). Clinically, precise and categorized interventions are required for varying sleep disorders. For patients with OSA suffering from nocturnal hypoxia, continuous positive airway pressure therapy maintains airway patency by generating positive intraluminal pressure. This treatment eliminates apneic and hypoxic events, severing pathophysiological cascades such as oxidative stress and sympathetic hyperactivation (de Lima et al., 2009). Conversely, for patients with chronic insomnia, long-term reliance on sedative-hypnotic medications is generally discouraged. Instead, cognitive behavioral therapy for insomnia (CBT-I) is recommended as a first-line treatment. CBT-I combines cognitive and behavioral strategies, including stimulus control, sleep restriction, and approaches to improve maladaptive sleep-related cognitions (Edinger et al., 2021). Effective sleep health management stabilizes nocturnal blood pressure fluctuations, thereby providing a highly favorable restorative environment for the cardiovascular system.

### 3.2 Pharmacotherapy

When lifestyle-modification strategies alone prove insufficient, guideline-directed pharmacotherapy remains essential for managing established cardiovascular risk and halting disease progression. Conventional agents including antihypertensive, lipid-lowering, antithrombotic, and heart-failure medications mitigate hemodynamic stress, atherosclerotic events, and recurrent cardiovascular complications (Virani et al., 2023; Netala et al., 2024). Contemporary pharmacotherapy has further advanced toward multidimensional risk reduction via combination approaches such as fixed-dose polypills (Castellano et al., 2022), together with therapies targeting broader cardiometabolic dysfunction (Ndumele et al., 2023). Notably, sodium-glucose cotransporter-2 (SGLT2) inhibitors and glucagon-like peptide-1 (GLP-1) receptor agonists exert cardiovascular benefits that extend beyond their primary glucose-lowering indications, including reduced heart-failure-related endpoints and fewer major adverse cardiovascular events (van der Aart-van der Beek et al., 2022; Lincoff et al., 2023). Nevertheless, cardiovascular events may still arise among individuals lacking conventional modifiable cardiovascular risk factors (Vernon et al., 2019). This observation highlights the demand for preventive strategies that address determinants outside the scope of standard risk-factor management.

### 3.3 Revascularization

For patients with clinically significant obstructive coronary artery disease, revascularization remains indispensable when lifestyle intervention and pharmacological therapy fail to control myocardial ischemia. Percutaneous coronary intervention rapidly restores coronary perfusion and serves as a critical strategy for managing acute coronary syndromes. By comparison, coronary artery bypass grafting remains a vital option for carefully selected patients with complex or multivessel coronary lesions (Thuijs et al., 2019; Byrne et al., 2023; Bytyçi et al., 2023; Virani et al., 2023). The choice between these two revascularization modalities relies on individualized evaluation of coronary anatomy, comorbid burden, procedural risks, and anticipated long-term clinical benefits. Importantly, revascularization targets only established anatomical lesions and cannot eliminate the upstream environmental and metabolic drivers that underpin residual cardiovascular risk.

### 3.4 Emerging therapeutic strategies

Emerging regenerative and molecular approaches are expanding therapeutic options for CVD beyond conventional pharmacological and revascularization strategies. Stem cell-derived products are being investigated for their potential to promote angiogenesis, tissue repair, and modulation of local inflammatory responses (Dehkordi et al., 2022; Jin and Liu, 2025), while biomaterial-based systems are being developed to support cardiac tissue repair and regeneration (Alamdari et al., 2022). Gene-based therapies, including genome and base-editing approaches, offer opportunities to target key genetic and metabolic drivers of CVD, although off-target effects, therapeutic durability, and long-term safety remain important translational challenges (Lee et al., 2022; Musolino et al., 2025). Targeted nanodelivery systems may further improve the tissue specificity and bioavailability of drugs, nucleic acids, and other therapeutic agents, but their clinical translation likewise depends on resolving issues related to biocompatibility, immunogenicity, and scalable manufacturing (Shariati et al., 2023; Rasekh et al., 2025). Collectively, these emerging approaches may improve the treatment of established cardiovascular pathology, whereas the prevention of preclinical disease susceptibility requires attention to early environmental and developmental determinants.

## 4 Intergenerational effects and early intervention of CVD

### 4.1 Epigenetic regulation and CVD

Given the persistent clinical burden and poor prognostic outcomes of CVD, contemporary research has decisively shifted its focus to early intervention, with intergenerational influences on offspring cardiovascular health emerging as an important frontier. As the concept “genetics loads the gun, but the environment pulls the trigger” suggests, environmental exposures influence epigenetic states (Münzel et al., 2021b). Parental exposure to environmental hazards, unhealthy lifestyles, and psychosocial stress may alter epigenetic regulation in germ cells, placental tissues, and developing offspring, thereby contributing to cardiovascular susceptibility in the next generation (**Figure 2**). Specifically, epigenetic modifications regulate gene expression mainly through deoxyribonucleic acid (DNA) methylation, histone modification, and non-coding ribonucleic acid (RNA) regulation (van der Harst et al., 2017). These processes are mediated by a variety of enzymes, including DNA methyltransferases (DNMTs), histone acetyltransferases (HATs), histone deacetylases (HDACs), and microRNA (miRNA) biosynthetic enzymes. Dysregulation of these enzymes may contribute to multiple pathological conditions, prominently including CVD.

**Figure 2:**
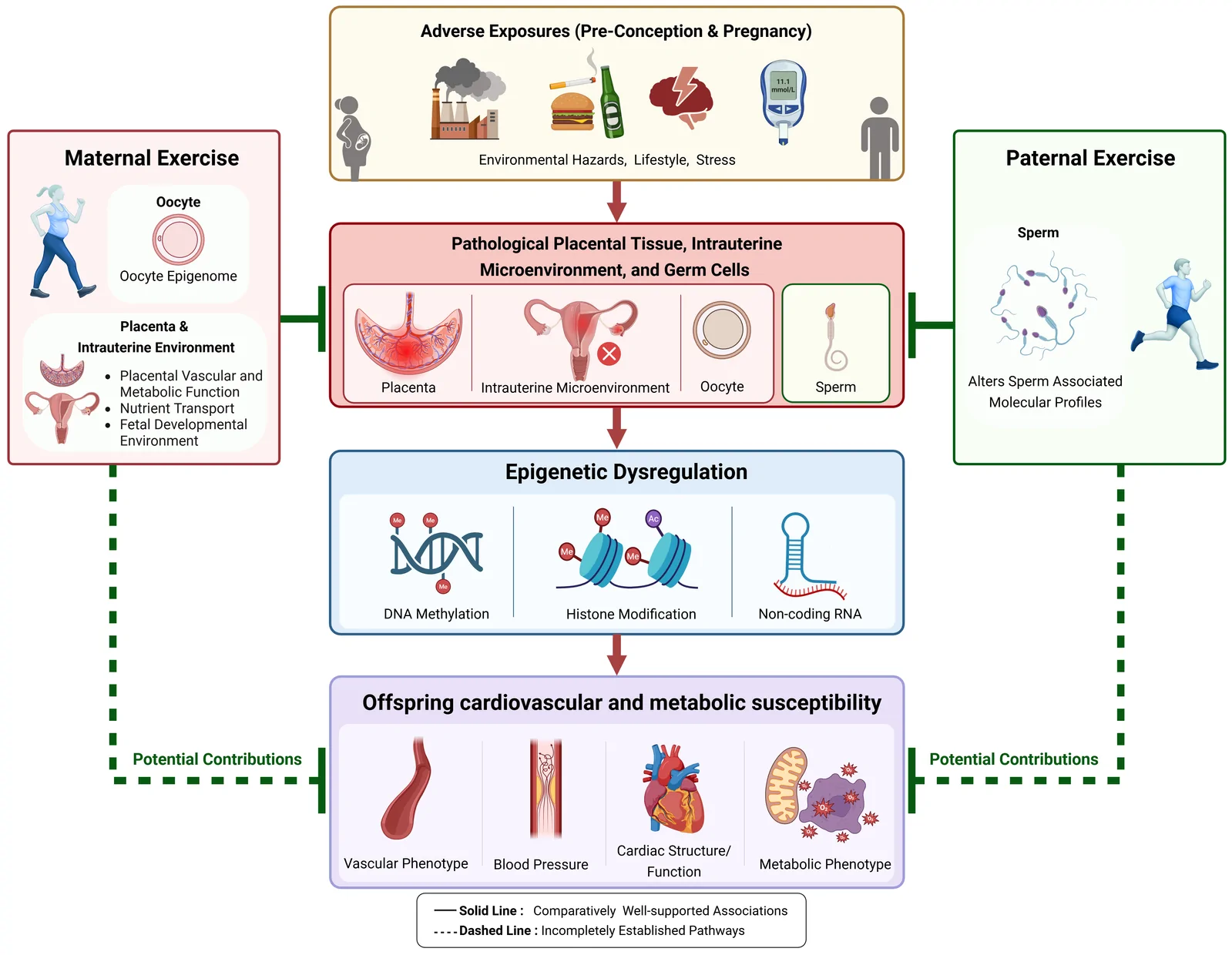
Epigenetic regulation of intergenerational cardiovascular risk and the protective effects of parental exercise. Adverse parental exposures before conception or during pregnancy may alter epigenetic regulation in placental tissue, intrauterine microenvironment, and germ cells, thereby contributing to cardiovascular and metabolic susceptibility in the next generation. Maternal exercise before and during pregnancy may favorably influence placental function and the intrauterine environment, whereas paternal pre-conception exercise may modify sperm-related biological characteristics. These exercise-associated adaptations may therefore contribute to favorable cardiovascular and metabolic phenotypes in offspring through potential epigenetic mechanisms. Created using biorender.com.

Epigenetic regulation represents one potential biological mechanism through which environmental exposures and metabolic stress may influence cardiovascular phenotypes. CVD arises from a complex interplay of genetic, environmental, cellular, and molecular factors, within which epigenetic regulation may contribute to disease development and therapeutic responses (Shi et al., 2022). Candidate-gene and epigenome-wide studies have identified DNA methylation patterns associated with CVD risk (Guarrera et al., 2015; Nakatochi et al., 2017). DNA methylation at cytosine-phosphate-guanine sites can influence transcriptional regulation, while histone modifications remodel chromatin structure and thereby alter gene expression (Lorenzen et al., 2012). Furthermore, non-coding RNAs participate in cardiovascular gene regulation and can interact with DNA methylation and histone-modification pathways (Wei et al., 2016).

### 4.2 Transmission pathways of adverse intergenerational effects

Epigenetic modifications constitute potential mechanisms through which parental exposures may shape offspring phenotypes. Parental environmental and health conditions have been associated with long-term offspring outcomes (Fleming et al., 2018). Maternal exposure to environmental hazards, unhealthy lifestyles, and psychosocial stress, together with paternal pre-conception factors, may alter reproductive and developmental epigenetic regulation, thereby contributing to intergenerational cardiovascular susceptibility (Batra et al., 2022).

#### 4.2.1 Environmental hazards and epigenetic programming

Prenatal exposure to environmental pollutants has been linked to epigenetic alterations in placental and offspring tissues. In human studies, maternal particulate matter 2.5 exposure during pregnancy correlates with reduced global DNA methylation in placental tissue (Janssen et al., 2013). Prenatal air-pollution exposure is also associated with altered newborn DNA methylation signatures and childhood cardiovascular risk markers including blood pressure and carotid intima-media thickness (Breton et al., 2016). These observations suggest that prenatal air pollution may modulate developmental epigenetic regulation and offspring cardiovascular susceptibility, potentially via altered DNA methylation.

Prenatal exposure to metals and metalloids is likewise associated with epigenetic modifications in offspring tissues. Within a prospective human birth cohort, prenatal arsenic exposure corresponded to altered DNA methylation patterns in umbilical-cord blood (Lv et al., 2025). Perinatal exposure to metals and metalloids has further been linked to offspring cardiovascular phenotypes and risk markers, most notably blood-pressure perturbations (Yim et al., 2022). These findings indicate that exposure-driven epigenetic changes may represent one pathway connecting the prenatal environment to cardiovascular susceptibility in offspring.

#### 4.2.2 Unhealthy lifestyles and metabolic factors in epigenetic programming

Maternal lifestyle and metabolic health during pregnancy are associated with epigenetic variation and health-related phenotypes in offspring. A systematic review of human randomized controlled trials and cohort studies reported associations between maternal diet, lifestyle, physical activity and differential DNA methylation as well as miRNA profiles, predominantly within placental and cord-blood tissues (Rasmussen et al., 2021). Nutritional imbalance before and during pregnancy can influence fetal growth and subsequent metabolic health (Marshall et al., 2021). Human studies have linked maternal obesity to differences in offspring cardiac structure and function, including reduced left-ventricular strain and increased interventricular-septal thickness in early postnatal life (den Harink et al., 2022). Gestational diabetes mellitus similarly confers adverse maternal and fetal outcomes and may promote unfavorable cardiometabolic phenotypes in offspring (Johns et al., 2018). Maternal metabolic dysfunction encompassing obesity, dyslipidemia and gestational diabetes is also associated with perturbed epigenetic regulation within the fetoplacental unit (Kerr et al., 2018).

Prenatal cigarette-smoke and alcohol exposure correlates with epigenetic and molecular changes in placental and offspring tissues. Clinical studies demonstrate that prenatal alcohol exposure alters placental DNA methylation, gene expression and vascular properties (Steane et al., 2021). Likewise, maternal smoking in pregnancy is associated with methylation differences at the insulin-like growth factor 2 (IGF2) differentially methylated region in umbilical-cord blood (Murphy et al., 2011). Such exposure-related molecular alterations may drive developmental programming and later-life disease susceptibility.

#### 4.2.3 Psychosocial stress and sleep disorders in epigenetic programming

Maternal psychosocial stress during pregnancy may affect offspring development and later disease susceptibility through maternal-placental-fetal endocrine and immune pathways (Entringer et al., 2015). In a human cohort comprising 661 mother-child dyads, prenatal perceived stress was associated with newborn-blood DNA methylation at nuclear receptor subfamily 3 group C member 1 (NR3C1), together with aggregated methylation patterns across NR3C1 and FKBP prolyl isomerase 5 (FKBP5), two genes central to HPA axis regulation (Jones et al., 2025). These findings suggest that prenatal psychosocial stress may reshape developmental stress-response circuits via epigenetic mechanisms.

Maternal sleep patterns during pregnancy may also relate to offspring epigenetic and metabolic phenotypes. In one prospective human birth cohort, delayed maternal sleep timing in late pregnancy was associated with differential cord-blood DNA methylation and higher adiposity among offspring at two years of age, with several methylation sites identified as putative mediators of these relationships (Meng et al., 2022). A recent review synthesizing human and rodent evidence further proposes that maternal sleep disorders in pregnancy may impair offspring metabolic health by remodeling the fetal developmental milieu, with epigenetic regulation among the candidate underlying mechanisms (Chen et al., 2025).

#### 4.2.4 Paternal factors and sperm epigenetic programming

Paternal pre-conception health and environmental exposures are associated with metabolic and cardiovascular phenotypes in offspring. A systematic scoping review integrating epidemiological, clinical and experimental work identified paternal obesity, diabetes, dietary imbalance, advanced paternal age, smoking and environmental chemical exposure as potential contributors to offspring cardiometabolic risk (Eberle et al., 2020). In pre-clinical models, sperm epigenetic alterations provide a plausible mechanism linking paternal exposures to offspring phenotypes. In a mouse model, chronic paternal psychological stress reshaped sperm DNA methylation and small non-coding RNA (sncRNA) profiles and was accompanied by reproductive, behavioral and metabolic phenotypes in subsequent generations (Zheng et al., 2021). A subset of sperm DNA methylation changes and associated phenotypes persisted into the F_2_ generation, offering pre-clinical evidence for transgenerational effects under these experimental conditions.

Paternal obesity and nutritional status are also linked to altered sperm quality and epigenetic signatures. Synthesized human and experimental data document obesity-related changes in sperm DNA methylation, chromatin organization and small-RNA profiles, which have been hypothesized as candidate mediators of offspring metabolic phenotypes (Raad et al., 2017; Billah et al., 2022). Nutritional evidence largely derived from animal models further indicates that high-fat, low-protein and other nutritionally imbalanced paternal diets can remodel sperm epigenetic features and modify offspring metabolic regulation (Dimofski et al., 2021). In a mouse low-protein-diet model, paternal diet altered cardiac and brain lipid profiles in offspring through mechanisms involving both sperm and seminal plasma (Furse et al., 2023). Collectively, these observations support sperm-mediated epigenetic regulation as one plausible pathway whereby paternal pre-conception exposures influence offspring cardiometabolic health.

### 4.3 Intergenerational effects of parental exercise

Pre-conception parental exercise and in-pregnancy maternal exercise represent potentially modifiable behavioral determinants that can shape metabolic and cardiovascular phenotypes in offspring. Current evidence indicates that maternal and paternal exercise operate through distinct yet complementary biological pathways: maternal exercise remodels the intrauterine and placental environment, whereas paternal exercise alters sperm-associated molecular profiles (Kusuyama et al., 2020; Félix-Soriano and Stanford, 2026). Epigenetic mechanisms including DNA methylation, histone modifications and sncRNAs have been proposed as candidate mediators of such intergenerational effects (Kusuyama et al., 2020; Félix-Soriano and Stanford, 2026). Recent data further show that responses to parental exercise vary across tissues and between male and female offspring, highlighting heterogeneity within intergenerational exercise-driven phenotypes (Félix-Soriano and Stanford, 2026).

#### 4.3.1 Human evidence for maternal-exercise-associated fetal cardiovascular adaptations

Human data suggest that maternal exercise during pregnancy is associated with shifts in fetal cardiovascular physiology without detectable impairment of uteroplacental circulation. Meta-analytic evidence shows that acute maternal exercise does not induce adverse perturbations in uterine or umbilical blood-flow parameters; regular in-pregnancy exercise, by contrast, correlates with lower resting fetal heart rate and reduced umbilical-artery vascular resistance (Skow et al., 2019). Consistent with these results, higher maternal physical-activity intensity in late pregnancy is associated with reduced fetal heart rate, while longer activity duration corresponds to elevated fetal heart-rate variability (May et al., 2012). Taken together, these observations indicate that maternal exercise can modulate fetal cardiovascular regulation while preserving uteroplacental hemodynamic function. Nevertheless, existing human evidence predominantly captures physiological endpoints measured during gestation and cannot independently establish whether these adaptations persist into postnatal life or are mediated by epigenetic mechanisms.

#### 4.3.2 Preclinical evidence: molecular and epigenetic programming by maternal exercise

Pre-clinical experimental evidence further suggests that maternal exercise programs offspring cardiovascular development via molecular and epigenetic regulation. Within the developing heart, maternal exercise is linked to enhanced mitochondrial biogenesis and dynamics, increased cytochrome c oxidase activity and adenosine triphosphate (ATP) production, alongside reduced hydrogen-peroxide levels, pointing to improved cardiac bioenergetic and redox homeostasis during fetal development (Chung et al., 2017). Non-coding-RNA-dependent regulation is also implicated: maternal exercise partially rescued diabetes-driven disturbances in fetal cardiac circular RNA (circRNA)-miRNA-messenger RNA (mRNA) interaction networks (Ye et al., 2023). More direct mechanistic evidence connecting exercise-triggered epigenetic modification to vascular function comes from hypertensive offspring models, where maternal exercise up-regulated vascular expression of the nicotinamide adenine dinucleotide (NAD^+^)-dependent deacetylase sirtuin 1 (SIRT1), lowered histone H3 lysine 9 (H3K9) acetylation at the NADPH oxidase 4 (Nox4) promoter, suppressed Nox4 transcription, and was accompanied by diminished oxidative stress and improved endothelium-dependent vasodilation (Zhang et al., 2023). Collectively, these findings support epigenetic regulation as a plausible mechanistic link between maternal exercise and offspring cardiovascular programming, though supporting evidence remains largely pre-clinical.

#### 4.3.3 The placenta as a mediator of maternal-exercise-driven fetal cardiovascular programming

The placenta constitutes a key interface through which maternal exercise modulates fetal cardiovascular development by reshaping placental vascular performance, nutrient handling and epigenetic regulation. Placental perfusion and substrate transfer establish the hemodynamic and metabolic milieu required for normal cardiac and vascular maturation, providing a biological rationale connecting maternal-exercise-induced placental adaptations to fetal cardiovascular programming (Umapathy et al., 2024). Among women with overweight or obesity, gestational exercise was associated with widespread placental DNA methylation changes, including hypomethylation at the carbonyl reductase 1 (CBR1) promoter, accompanied by elevated CBR1 expression and reduced oxidative-stress indices (Wang et al., 2024). Maternal exercise may also improve placental vascular and metabolic function. In obese mice, exercise enhanced placental vascularization together with changes in vascular endothelial growth factor (VEGF)-related markers and activation of AMP activated protein kinase (AMPK), mechanistic target of rapamycin complex 1 (mTORC1), and insulin signaling pathways (Son et al., 2019). Human studies further link prenatal physical activity to altered placental sodium coupled neutral amino acid transporter 2 (SNAT2) and fatty acid transport protein 4 (FATP4) expression, pointing to exercise-sensitive nutrient transport at the maternal-fetal boundary (Brett et al., 2015; Hutchinson et al., 2020). Taken together, these data identify the placenta as a plausible biological intermediate between maternal exercise and fetal cardiovascular programming, in which vascular, metabolic and epigenetic adaptations jointly shape the intrauterine environment.

#### 4.3.4 Paternal pre-conception exercise and germline-mediated intergenerational effects

Relative to maternal exercise, evidence describing intergenerational effects of paternal exercise remains more limited and centers predominantly on pre-conception remodeling of the male germline. In mouse studies, paternal exercise mitigated high-fat-diet-provoked impairments in offspring glucose metabolism and adiposity while reprogramming sperm small-RNA profiles, supporting sperm-borne molecular signals as candidate mediators of exercise-related offspring phenotypes (Stanford et al., 2018). More recent mechanistic work reinforces this model, demonstrating that exercise-elicited sperm-miRNA changes can reshape early-embryonic transcriptional landscapes and recapitulate improved endurance and metabolic function in offspring following sperm small-RNA transfer (Yin et al., 2025). Human evidence, however, is currently largely restricted to alterations within the paternal germline itself: exercise training remodels global and genome-wide DNA methylation profiles in human sperm, yet it remains unknown whether such modifications translate to altered cardiovascular phenotypes in offspring (Denham et al., 2015). Accordingly, the male germline is responsive to paternal exercise, although evidence connecting these exercise-induced epigenetic alterations to offspring cardiovascular outcomes remains sparse and mostly pre-clinical.

#### 4.3.5 Mechanistic framework: metabolite-sensitive epigenetic regulation

Mechanistically, parental exercise may couple systemic physiological adaptations to offspring cardiovascular programming via metabolite-sensitive epigenetic regulation. Exercise remodels the circulating milieu through exerkines and metabolic signals, providing a potential route through which parental physiological states may be communicated to reproductive and developing tissues (Son et al., 2020; García-Giménez et al., 2024). In a mouse model, maternal exercise increased apelin in maternal and fetal circulation and enhanced α-KG-associated DNA demethylation of the Prdm16 promoter in fetal brown adipose tissue (Son et al., 2020), illustrating how an exercise-responsive circulating mediator may engage epigenetic regulation during development. More broadly, exercise-induced changes in metabolites and cofactors may influence epigenetic enzymes, including DNMTs, TET enzymes, HATs, and HDACs (García-Giménez et al., 2024). During early germ-cell development, α-KG serves as a cofactor for TET enzymes and Jumonji C-domain histone demethylases, linking cellular metabolism to DNA and histone demethylation (Verdikt and Allard, 2021), whereas lactate can modulate HDAC activity in experimental systems (Latham et al., 2012). These metabolite-sensitive mechanisms may provide a plausible upstream framework for the sperm DNA methylation and small-RNA changes observed following paternal exercise (Denham et al., 2015; Stanford et al., 2018; Yin et al., 2025), although direct links between exercise-induced systemic signals and epigenetic-enzyme activity in germ cells remain incompletely established. Importantly, cardiovascular tissues also undergo exercise-associated epigenetic reprogramming, and these changes correlate with improved vascular function in adult offspring (Zhang et al., 2023).

Overall, available evidence suggests that maternal exercise can program offspring cardiovascular development via intrauterine and placental adaptations, whereas paternal exercise acts primarily through sperm-associated molecular and epigenetic remodeling. Epigenetic regulation therefore represents a plausible mechanistic bridge between parental exercise and intergenerational cardiovascular programming, yet direct human evidence documenting long-term cardiovascular consequences in offspring remains limited.

## 5 Conclusions and future perspectives

CVD remain a global public health challenge, driven by a rapidly evolving pathogenic landscape. The modern cardiovascular exposome, comprising environmental hazards, sedentary lifestyles, and psychosocial stress, synergistically disrupts physiological homeostasis. To counteract this multidimensional damage, contemporary clinical management has transitioned into an integrated framework. By combining lifestyle optimization, multi-target pharmacotherapy, and emerging biotechnologies, current strategies offer robust pathways to delay disease progression. Nevertheless, most current interventions focus on the middle and late stages after disease onset. Therefore, shifting the intervention window forward has become an important direction for cardiovascular prevention.

Addressing this need, modern research has extended the timeline of disease prevention to the pre-conception and gestational stages, highlighting intergenerational influences on offspring cardiovascular health. Epigenetic regulation may provide an important biological link between parental environmental exposures and cardiovascular susceptibility in offspring. Adverse parental exposures may alter epigenetic regulation in germ cells, placental tissues, and developing offspring, whereas parental exercise may favorably influence these processes through complementary maternal and paternal pathways. Maternal exercise may modify placental vascular, metabolic, and epigenetic adaptations and thereby influence the intrauterine environment and fetal cardiovascular development, while paternal pre-conception exercise may alter sperm-associated molecular and epigenetic profiles. Exercise-responsive circulating factors and metabolites may also interact with metabolically sensitive epigenetic machinery, providing a potential mechanism linking parental physiological adaptations to developmental programming. Current human evidence primarily supports associations between maternal exercise and fetal or placental adaptations, together with exercise-associated epigenetic changes in human sperm, whereas direct mechanistic evidence linking parental exercise-induced epigenetic alterations to persistent cardiovascular phenotypes in offspring remains predominantly preclinical.

The future prevention and management of CVD should therefore increasingly adopt a life-course perspective. Further research is required to clarify the complex interactions within the cardiovascular exposome and, importantly, to determine how parental exposures and exercise-associated epigenetic adaptations influence long-term cardiovascular health in offspring. Clinical research should further evaluate these intergenerational determinants through longitudinal human studies extending from the pre-conception and gestational periods into later life. By advancing the preventive window and emphasizing the health of both parents, intergenerational approaches may complement conventional cardiovascular risk management and provide new opportunities for earlier prevention of CVD.

## Glossary

ACE2: Angiotensin-converting enzyme 2
α-KG: α-Ketoglutarate
AMPK: AMP-activated protein kinase
ARNIs: Angiotensin receptor-neprilysin inhibitors
ATP: Adenosine triphosphate
BMAL1: Brain and muscle ARNT-like 1
CABG: Coronary artery bypass grafting
CBR1: Carbonyl reductase 1
CBT-I: Cognitive Behavioral Therapy for Insomnia
CLOCK: Circadian Locomotor Output Cycles Kaput
CO: Carbon monoxide
COVID-19: Coronavirus disease 2019
CVD: Cardiovascular disease
DASH: Dietary Approaches to Stop Hypertension
DNA: Deoxyribonucleic acid
DNMTs: DNA methyltransferases
eNOS: Endothelial nitric oxide synthase
FATP4: Fatty acid transport protein 4
FKBP5: FKBP prolyl isomerase 5
GLP-1: Glucagon-like peptide-1
H3K9: Histone H3 lysine 9
HATs: Histone acetyltransferases
HDACs: Histone deacetylases
HIIT: High-intensity interval training
HPA: Hypothalamic-pituitary-adrenal
IGF2: Insulin-like growth factor 2
IL-6: Interleukin-6
miRNA: MicroRNA
mTORC1: Mechanistic target of rapamycin complex 1
NAD⁺: Nicotinamide adenine dinucleotide
NF-κB: Nuclear factor kappa B
Nox4: NADPH oxidase 4
NR3C1: Nuclear receptor subfamily 3 group C member 1
OSA: Obstructive sleep apnea
PCI: Percutaneous coronary intervention
RNA: Ribonucleic acid
ROS: Reactive oxygen species
SAM: Sympathetic-adrenal-medullary
SARS-CoV-2: Severe acute respiratory syndrome coronavirus 2
SGLT2: Sodium-glucose cotransporter 2
SIRT1: Sirtuin 1
SNAT2: Sodium-coupled neutral amino acid transporter 2
sncRNA: Small non-coding RNA
TET: Ten-eleven translocation
TLR4: Toll-like receptor 4
VEGF: Vascular endothelial growth factor
